# Clinical impact of ^18^F-FDG PET/CT on initial staging and therapy planning for breast cancer

**DOI:** 10.3892/etm.2012.659

**Published:** 2012-08-09

**Authors:** BENGUL GUNALP, SEMRA INCE, ALPER OZGUR KARACALIOGLU, ASLI AYAN, OZDES EMER, ENGIN ALAGOZ

**Affiliations:** Department of Nuclear Medicine, Gulhane Military Medical Academy and Faculty, 06018 Ankara, Turkey

**Keywords:** ^18^F-FDG, PET, CT, breast cancer, primary staging, therapy planning

## Abstract

The purpose of this study was to determine the clinical significance of ^18^F-FDG PET/CT on initial staging and therapy planning in patients with invasive breast cancer. One hundred and forty-one consecutive, biopsy proven preoperative and 195 postoperative high-risk breast cancer patients who were referred for PET/CT for initial staging were included in this retrospective study. The clinical stage had been determined by conventional imaging modalities prior to the PET/CT scan. Of the 141 examined preoperative patients, 19 had clinical stage I (T1N0), 51 had stage IIA (12 T2N0 and 39 T1N1), 49 had stage IIB (2 T3N0 and 47 T2N1), 12 had stage IIIA (11 T3N1, 1 T2N2), 2 had stage IIIB (2 T4N1) and 8 had stage IV. PET/CT modified the staging for 26% of stage I patients, 29% of stage IIA patients, 46% of stage IIB patients, 58% of stage IIIA patients and 100% of stage IIIB patients. PET/CT scans detected extra-axillary regional lymph nodes in 14 (9.9%) patients and distant metastasis in 41 (29%) patients. PET/CT scans detected multifocal lesions in 30 (21%) patients, multicentric lesions in 21 (14%) patients and malign foci in the contralateral breast (bilateral breast cancer) confirmed by biopsy in 5 (3.5%) patients. Of the examined 195 postoperative patients PET/CT detected axillary lymph nodes in 22 (11%) patients, extra-axillary regional lymph nodes in 21 (10%) patients and distant metastasis in 24 (12%) patients. PET/CT findings altered plans for radiotherapy in 22 (11%) patients and chemotherapy was adapted to the meta-static diseases in 24 (12%) patients. PET/CT was revealed to be superior to conventional imaging modalities for the detection of extra-axillary regional metastatic lymph nodes and distant metastases. These features make PET/CT an essential imaging modality for the primary staging of invasive breast cancer, particularly in patients with clinical stages II and III.

## Introduction

Breast cancer is the most common cancer in women and the second leading cause of cancer mortality in women (1). Accurate diagnosis and staging are essential for the selection of the most appropriate therapeutic strategy and major determinants of patient prognosis and survival (2).

A systematic work-up for operable invasive breast carcinoma has usually been performed by physical examination, bilateral mammogram and ultrasound with or without breast MRI. In the case of operable stage IIIA (T3N1M0), additional imaging, including bone scanning (BS), abdominal and pelvic CT (or ultrasound or MRI), and chest imaging, may be used. The significance of PET/CT in initial staging of breast carcinoma has not yet been well-defined in routine clinical practice. Clinicians usually refer patients for PET/CT scan when conventional imaging studies are equivocal, suggestive or considered as ‘high risk’ according to patients’ histological and surgical manifestations. Several studies show that PET/CT provides important information in patients with stage II or III breast carcinoma with the detection of unknown lymph node metastases outside axillary levels I and II (infraclavicular, supraclavicular and internal mammary nodes) and the detection of occult distant metastases (3–8). However, this study included patients with stage II or III breast cancer. In this retrospective study, we also included stage I patients of invasive breast carcinoma (invasive ductal, invasive lobular or mix type) with high histological grade (grade II–III) and postoperative patients for whom clinical work-up had been performed by conventional imaging modalities (CIM; mammogram, ultrasound, chest X-ray, breast MRI, BS, abdominal and pelvic CT or ultrasound or MRI) prior to surgery.

## Materials and methods

In this retrospective study we included 141 consecutive newly diagnosed, histological high grade (grade II–III) preoperative patients (mean age 47 years, range 28–78 years) and 195 postoperative high risk breast cancer patients (mean age 48 years, range 25–75 years) who were referred to PET/CT for initial staging. Clinical stage had been determined by physical examination, mammography, ultrasound of the breast and axilla, and the breast MRI. The clinical stage III patients underwent a conventional imaging work-up with BS, abdominal and pelvic CT (or ultrasound or MRI), and chest imaging. T and N clinical scores were evaluated according to the American Joint Committee on Cancer (AJCC) classification (9).

No patients in this series had received chemotherapy or radiotherapy prior to PET/CT examination. The patients fasted for 6 h as the blood glucose level had to be less than 150 g/ml. ^18^F-FDG (5 MBq/kg) was intravenously injected into the arm opposite to the tumor using a venous line to prevent extravasation. Imaging was performed 60 min after the injection on a PET/CT scanner (GE Discovery 690 PET/CT).

The PET/CT scans were interpreted by 2 nuclear medicine specialists. If the interpretation differed, consensus was reached with the aid of a third reader. The readers relied on visual assessment of PET images (a well-defined focus, with uptake clearly higher than surrounding background). The location of hypermetabolic lymph nodes on the PET/CT image was noted according to the AJCC seventh classification (9).

For distant metastases, form and intensity of ^18^F-FDG uptake and CT findings were considered altogether. ^18^F-FDG uptake corresponding to degenerative findings on the underlying CT scan (e.g. on facet articulation) and uptake in a rib fracture in a patient with a history of trauma were considered benign. However, high uptake on a classic area of metastasis (e.g. body of a vertebra, pedicle, long bone) was considered malignant even if the CT scan revealed subtle or no change, in agreement with the well-known high sensitivity of ^18^F-FDG PET, compared with CT, for early bone marrow involvement (10). For lung evaluation, we considered any pulmonary nodules with high ^18^F-FDG uptake or the presence of multiple small round nodules on the CT part as suggestive (even in the absence of an increase in ^18^F-FDG uptake).

PET/CT findings considered to be suggestive of malignancy were assessed using surgery, biopsy results or patient follow-up. For bone foci, MRI was performed instead of biopsy. We considered modification of stage resulting from findings of distant metastasis or lymph node involvement outside classic areas of axillary dissection, with an impact on treatment management. Staging using PET/CT was compared with that of the conventional modalities.

## Results

### Primary staging of preoperative patients

*Stage I.* Nineteen patients had clinical stage I (T1N0). All of the primary tumors had clear ^18^F-FDG uptake [median maximum standardized uptake value (SUVmax), 5.7; range, 1.2–13.7].

**Figure 1:**
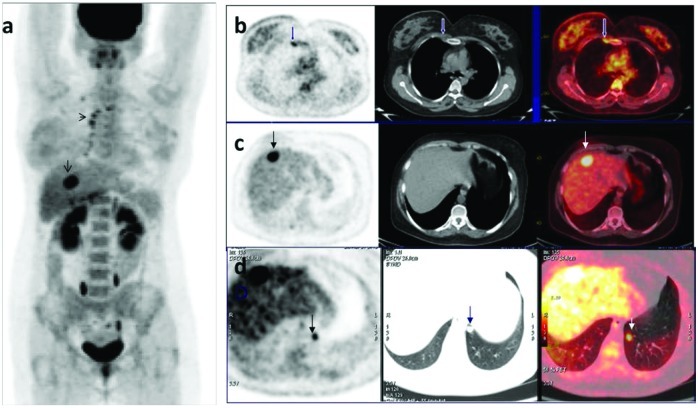
Postoperative PET/CT scan of a patient whose preoperative clinical stage had been determined by conventional imaging modalities as stage I (T1N0M0). The patient underwent right breast-conserving surgery and SLNB. SLNB was negative. Postoperative pathology revealed ER (−), PR (−), HER-2 (−) invasive ductal carcinoma. The patient was classified as high risk according to histopathological findings and referred to PET/CT exam for the evaluation of possible metastatic spread. (a) Maximum intensity projection PET, (b–d) axial PET, CT and PET/CT demonstrate multiple (a and b) internal mammary lymph node involvements, (c) liver and (d) lung metastases. The patient was re-staged as stage IV and metastatic treatment was started. SLNB, sentinel lymph node biopsy.

PET/CT increased the stage of 5 patients (26%). Uptake in axillary lymph nodes in 2 patients, internal mammary lymph nodes in 2 patients and bone metastases in one patient were detected by PET/CT (Fig. 1).

*Stage IIA.* Fifty-one patients had clinical stage IIA (12 T2N0M0 and 39 T1N1M0). All primary tumors had ^18^F-FDG uptake (T2N0M0 median SUVmax, 8.7; range, 2.8–15.8; T1N1M0 median SUVmax, 6.1; range, 1.8–21.7).

PET/CT increased the stage of 4 patients (33%) in the T2N0 group. In 2 patients axillary level II lymph nodes which were not detected by ultrasonographic (USG) imaging, in one patient bone metastases which were not detected by BS (confirmed by follow-up) and in one patient liver metastases were not detected by contrast enhanced CT (CE-CT; confirmed by MRI), were identified.

PET/CT increased the stage of 11 patients (28%) in the T1N1 group. Exta-axillary lymph node metastases in 3 patients (infraclavicular and supraclavicular lymph node metastases in 3 patients) and distant metastases in 8 patients (5 bone, 1 bone and pleura, 1 bone and lung and 1 contralateral breast) were detected.

*Stage IIB.* Forty-nine patients had clinical stage IIB (2 T3N0 and 47 T2N1). All primary tumors had ^18^F-FDG avidity (in T3N0 group median SUVmax, 12.2; range, 9.5–14.6; in T2N1 group median SUVmax, 9.4; range, 2.3–20.9).

PET/CT changed the stage in 23 patients (48%). Infraclavicular (level III) uptake in 2 patients and internal mammary uptake in 4 patients initially classified as T2N1 were detected. These patients were reclassified as N3b (stage IIIC). ^18^F-FDG uptake which was suggestive of distant metastasis was observed in 24 women: 11 with bone, 2 with liver (confirmed by MRI), 1 with both bone and surrenal, 3 with bone and liver metastases and 1 with pleural involvement.

*Stage IIIA.* Twelve patients had clinical stage IIIA (11 T3N1 and 1 T2N2). All primary tumors exhibited ^18^F-FDG uptake (median SUVmax, 9.1; range, 3.2–28.5).

PET/CT changed the staging in 7 (58%) patients. PET/CT revealed N3 lymph nodes (infra- or supraclavicular or internal mammary) in 3 (25%) patients and uptake suggestive of distant metastases in 4 (33%) patients. Sites of involvement in the 4 patients with distant lesions were bone (n=1), bone and liver (n=2) and contralateral breast (n=1).

*Stage IIIB.* Two patients had clinical stage IIIB (2 T4N1). The primary tumor showed ^18^F-FDG uptake (median SUVmax, 8.4; range, 6.8–10).

PET/CT changed the staging in both patients (100%). PET/CT detected internal mammary lymph nodes and distant metastases in 2 patients (100%). There were bone metastases in one and both bone and lung metastases in the other patient.

PET/CT detected multifocal lesions in 30 (21%) patients, multicentric lesions in 21 (14%) patients and malign foci in the contralateral breast (confirmed by biopsy) in 5 (3.5%) patients.

In the detection of metastatic subcentimetric pulmonary nodules with low ^18^F-FDG uptake, the CT component of PET/CT increased sensitivity. By contrast, the PET component of PET/CT revealed malign pleural invasion and effusion, and adrenal and liver metastases which were equivocal in CT.

PET/CT detected liver metastasis in 13 patients, of which only 5 had evidence by CE-CT.

PET/CT revealed true-positive bone metastases in 35 patients whereas BS revealed metastases in only 21 (61%). In all 14 patients with negative BS results, MRI and follow-up confirmed bone involvement. Five of these 14 women had additional visceral metastases.

PET/CT changed the staging, with impact on therapeutic management in 15% (3/19) of stage I patients, 25% (13/51) of stage IIA patients, 48% (24/49) of stage IIB patients, 58% (7/12) of stage IIIA patients and 100% (2/2) of patients with stage IIIB due detection of extra-axillary and distant metastasis. The planning for radiotherapy was modified according to PET/CT results to encompass the internal mammary basin. Chemotherapy was adapted to the metastatic diseases and certain bone lesions were treated by radiation therapy (Tables I and II).

### Postoperative patients

Of the examined 195 postoperative patients, PET/CT detected residual tumor foci in the original breast which reflects insufficient surgery in 18 patients (9%). Mastectomy should have been performed on these patients instead of breast-conserving surgery.

PET/CT detected ipsilateral axillary lymph nodes in 22 (11%) patients, extra-axillary regional lymph nodes in 21 (10%) patients (10 internal mammary, 4 infraclavicular, 9 supraclavicular, 11 mediastinal, 4 jugular) and distant metastasis in 24 (12%) patients (18 bone, 2 liver, 4 lung, 1 pleura and 2 adrenal). None of these metastatic sites had been detected by conventional imaging modalities prior to surgery.

Additional PET/CT findings changed radiotherapy planning in 22 (11%) patients and chemotherapy was adapted to the metastatic diseases in 24 (12%) patients (Table III).

Five of eighteen postoperative patients whom PET/CT showed bone metastases had negative preoperative BS and none underwent BS following post surgical PET/CT scan since all patients had additional nodal or visceral metastasis and the majority were confirmed by MRI.

False-positive findings in articular regions which belongs usually to inflammatory or degenerative changes in the traumatic fracture sites were differentiated from malign involvements by the CT component of PET/CT.

## Discussion

There are several studies which show that PET/CT provides important information on patients with stage II or III breast carcinoma with the detection of unknown lymph node metastases outside axillary levels I and II and detection of occult distant metastases (3–8). Previous studies suggest that PET/CT likely has no role in initial staging in patients with T1 (≤2 cm) tumors since the sensitivity to detect the primary tumor and axillary involvement is too low and the probability of finding distant metastases is also low. These results are explained by the various histopathology and size of the tumors. Lower sensitivity has been reported in more differentiated and slow-growing tumors and in noninvasive breast cancer, whereas improved performance has been demonstrated for the detection of primary invasive breast cancer with an overall sensitivity, specificity and accuracy of 90, 93 and 92%, respectively (11). In our retrospective study all patients had grades II and III invasive breast carcinoma. All 58 patients with T1 tumor (19 T1N0, 39 T1N1) had good FDG uptake (median SUVmax, 5.9) and were detected by PET/CT (sensitivity and specificity 100%). PET/CT also detected internal mammary lymph node involvements in 2 patients and early bone marrow metastases in 1 patient with stage I disease. These findings prove that invasive breast cancer is a systemic disease and even early breast cancer may give rise to metastases which were not detected previously by routine clinical work-up.

In this study PET/CT was demonstrated to be more sensitive in detecting multifocal lesions than the combination of mammography and ultrasonography. PET/CT detected multifocal lesions in 30 (21%) and multicentric lesions in 21 (14%) preoperative patients. In the postoperative group, PET/CT detected residual tumor foci in 18 (9%) patients. Ultrasonography and mammography combination had not identified these lesions and patients underwent breast-conserving surgery instead of mastectomy.

PET/CT identified axillary levels I and II lymph node involvements in 109 (55%) preoperative patients. In 22 (11%) postoperative patients PET/CT demonstrated increased metabolic activity in the axillary lymph nodes which have typical morphology for the metastases (round shape, loss of fatty hilus). This postoperative group revealed that sentinel lymph node biopsy (SLNB) may not detect macroscopic axillary metastatic lymph nodes in at least 10% of high risk patients. This false-negative rate of SLNB is too high in this high risk population with high prevalence of axillary lymph node metastases (≥40%) and may be due to massive invasion of a sentinel node that has lost its functional capacity of phagocytosis and is detected by PET/CT. By identifying 50% or more of cases of clinically occult lymph node disease in the axilla, PET/CT reduces the risk linked to false-negative SLNB. Since the positive predictive value is high in patients with positive FDG PET/CT in the axilla (82%), axillary lymph node dissection (ALND) may be performed directly without SLNB (12–14).

Detecting lymph node involvement in levels or basins other than those addressed by routine ALND may have a major impact on treatment strategies. Numerous studies suggest that FDG PET outperforms conventional imaging in detecting involvement in high-level axillary (level III) as well as in supraclavicular and internal mammary lymph nodes (3,4,6,15,16) PET/CT is particularly appealing compared to PET alone as it provides the precise location of involved nodes (4,6,16). In addition, false positives due to muscular and brown fat uptake are avoided. In this study PET/CT revealed unsuspected infraclavicular node involvement (N3a) in 11 (7.8%) preoperative and in 4 (2%) postoperative patients, and supraclavicular node involvement (N3c) in 8 (5.6%) preoperative and in 9 (4.6%) postoperative patients. The differentiation of level III axillary lymph nodes (infraclavicular lymph nodes) is important since axillary clearance usually includes levels I and II nodes only. Surgical studies have demonstrated that level III node involvement (N3a) results in a poorer prognosis (17), for which a subsequently modified surgical approach is useful (4). The detection of extra-axillary involvement (supraclavicular and/or internal mammary lymph nodes) is also extremely useful in delineating the radiotherapy target zone or schedule surgery. Patients with supraclavicular lymph nodes (N3c) may receive more intensive treatment, combining induction chemotherapy, surgery, postsurgical chemotherapy and irradiation, which improves the disease-free survival and overall survival (18). In addition, visualization of an internal mammary hot node by initial PET/CT may lead to a decision on surgery and/or radiotherapy (4).

The findings of metastasis on the initial work-up markedly changes treatment approaches. PET (19–21), particularly PET/CT (4,10,16), outperforms classic modalities to detect occult metastases. In this retrospective study, on 195 postoperative high risk breast cancer patients, PET/CT revealed distant metastases in 24 (12%) patients whose metastatic involvement was not detected by conventional imaging during the preoperative work-up.

Fuster *et al* (6) studied 60 consecutive patients with breast cancer stage IIB or higher. PET/CT sensitivity and specificity in detecting distant metastasis were 100 and 98%, respectively, vs. 60 and 83% for conventional work-up (contrast-enhanced chest CT, liver ultrasonography, 99 mTc-HDP BS). It is well-known that PET outperforms BS for the detection of lytic metastases and for early intra-medullary involvement, but has lower sensitivity in cases of pure osteoblastic lesions (22). PET/CT is also highly sensitive in detecting pleural, mediastinal, abdominal and pelvic metastases. PET performs well to assess lung nodules larger than 1 cm, but its sensitivity is low in smaller lesions, due to partial volume effect and respiratory motion. Careful interpretation of the CT images may depict small pulmonary nodules that do not present as hot spots on PET.

In conclusion, this retrospective study clearly showed that PET/CT appears to outperform conventional modalities in the initial work-up of invasive breast cancer patients. PET/CT allows diagnosis of infraclavicular, supraclavicular and internal mammary node involvement and may detect occult distant metastases. Additional PET/CT findings (findings of N3 disease or distant disease) to conventional imaging modalities may cause substantial change in the management of patients. In particular, PET/CT findings in postoperative patients clearly showed that clinical work-up by conventional imaging is insufficient for initial staging of invasive breast carcinoma and PET/CT should be used as a first-line test for high risk patients.

## Figures and Tables

**Table I:** Clinical stages of preoperative patients.

Clinical stage/T, N, M	No. of patients
Stage I	
T1N0	19
Stage IIA	
T2N0	12
T1N1	39
Total	51
Stage IIB	
T3N0	2
T2N1	47
Total	49
Stage IIIA	
T3N1	11
T2N2	1
Total	12
Stage IIIB	
T4N1	2
Stage IV	
Any T, any N, M1	8
Total	141

**Table II:** Impact of ^18^F-FDG PET/CT results in preoperative patients [number of patients (% per-patient basis)].

Variable	Stage I	Stage IIA	Stage IIB	Stage IIIA	Stage IIIB
No. of patients	19	51	49	12	2
Overall stage modification with impact on therapeutic management	3 (15%)	13 (25%)	23 (48%)	7 (58%)	2 (100%)
Detection of unknown extra-axillary lymph node metastases	2 (10%)	3 (5.8%)	6 (12.2%)	4 (33.3%)	2 (100%)
Internal mammary	2	-	4	1	2
Infraclavicular	-	2	2	1	-
Supraclavicular	-	1	-	1	-
Mediastinal	-	-	-	1	-
Detection of unsuspected distant metastases	1 (5%)	10 (19.6%)	20 (40%)	7 (58%)	2 (100%)
Bone metastases	1	8	13	4	2
Liver metastases	-	1	3	2	-
Lung metastases	-	1	1	1	1
Other sites (surrenal, pleura)	-	1	3	-	-

**Table III:** ^18^F-FDG PET/CT findings in postoperative patients (% per-patient basis).

Variable	No. of patients
Detection of residual tumor	18 (9.2%)
Detection of axillary lymph nodes involvement which were not detected by CIM and SLNB prior to surgery	22 (11%)
Detection of unknown extra-axillary node metastases	21 (10%)
Internal mammary	10
Infraclavicular	4
Supraclavicular	9
Mediastinal	11
Jugular	4
Detection of unsuspected distant metastases	24 (11%)
Bone	18
Liver	2
Lung	4
Pleura	1
Adrenal	2
Modification in post-operative treatment plan	46 (23%)
Radiotherapy planning	21 (10%)
Chemotherapy for metastatic disease	24 (11%)

CIM, conventional imaging modalities; SLNB, sentinel lymph node biopsy.
